# Optical Properties and Mechanical Modeling of Acetylated Transparent Wood Composite Laminates

**DOI:** 10.3390/ma12142256

**Published:** 2019-07-13

**Authors:** Kyle E. O. Foster, Kristen M. Hess, Garret M. Miyake, Wil V. Srubar

**Affiliations:** 1 Materials Science and Engineering Program, University of Colorado Boulder, UCB 596, Boulder, CO 80309-0596, USA; 2 Department of Civil, Environmental, and Architectural Engineering, University of Colorado Boulder, ECOT 441 UCB 428, Boulder, CO 80309-0428, USA; 3 Department of Chemistry, Colorado State University, Center Ave, Fort Collins, CO 80523, USA

**Keywords:** transparent wood, laminates, interfacial modification, acetylation, mechanical modeling

## Abstract

Transparent wood composites (TWCs) are a new class of light-transmitting wood-based materials composed of a delignified wood template that is infiltrated with a refractive- index-matched polymer resin. Recent research has focused primarily on the fabrication and characterization of single-ply TWCs. However, multi-ply composite laminates are of interest due to the mechanical advantages they impart compared to the single ply. In this work, 1- and 2-ply [0°/90°] TWC laminates were fabricated using a delignified wood template (C) and an acetylated delignified wood template (AC). The optical and mechanical properties of resultant C and AC TWC laminates were determined using ultraviolet-visible spectroscopy (UV-Vis) and tensile testing (5× replicates), respectively. In addition, the ability of classical lamination plate theory and simple rule of mixtures to predict multi-ply tensile modulus and strength, respectively, from ply-level mechanical properties were investigated and are reported herein. Experimental results highlight tradeoffs that exist between the mechanical and optical responses of both unmodified and chemically modified TWCs. Template acetylation reduced the stiffness and strength in the 0° fiber direction by 2.4 GPa and 58.9 MPa, respectively, compared to the unmodified samples. At high wavelengths of light (>515 nm), AC samples exhibited higher transmittance than the C samples. Above 687 nm, the 2-ply AC sample exhibited a higher transmittance than the 1-ply C sample, indicating that thickness-dependent optical constraints can be overcome with improved interfacial interactions. Finally, both predictive models were successful in predicting the elastic modulus and tensile strength response for the 2-ply C and AC samples.

## 1. Introduction

Transparent wood composites (TWCs) are a new class of engineered composite materials that capitalize on the cellular architecture of delignified wood templates infiltrated with refractive index-matched polymers to produce tough composites with a unique ability to transmit light. TWCs were originally developed in the early 1990s to study the detailed cellular structure of wood but have recently garnered attention as a structural material due to useful characteristics, such as good mechanical properties, transparency, and biorenewability [1,2,3]. For example, TWCs have been explored as alternatives to silica glass in window applications, where lower thermal conductivities, non-brittle failure modes, diffuse illumination, and lower environmental footprints are desirable [4,5,6].

Many challenges, including haze and thickness, currently exist regarding the fabrication and implementation of TWCs. Increased haze occurs in TWCs because of (1) the chemical interfacial heterogeneity between cellulosic constituents and the resin, (2) the difficulty in precise matching of refractive indices, and (3) natural defects in the delignified wood template that may restrict polymer infiltration [6]. Thickness is another challenge for TWCs. Fabrication of thick (>5 mm), uniform TWC samples that maintain optical transparency is an active area of research. Past studies have investigated the use of various types of woods, polymers, and fiber orientations to overcome thickness limitations without compromising optical transmittance [3,4,5,6,7,8,9,10]. High transmittance is best achieved in TWCs when wood template fibers are aligned with the direction of light propagation. However, using the delignified wood template as a waveguide in such a way compromises the structural integrity of the TWC; wood is not as mechanically stiff or strong in its tangential or radial directions as compared to the longitudinal [3]. In terms of substrates, most TWCs have been fabricated from balsa wood, a highly porous substrate that facilitates polymer infiltration [2,5]. A study using a delignified wood template from balsa wood and poly(methyl methacrylate) (PMMA) reported a transmittance of 60% and haze of 78% at a sample thickness of 3 mm [5]. Other wood substrates, such as birch, beech, bass, and poplar, have also been used. However, no TWCs from these substrates achieved thicknesses >2 mm without lamination or mechanically compromising the TWCs [3,4,5,7,8,9,10].

Methods, such as chemical compatibilization and composite lamination, have been investigated to improve the mechanical properties while ensuring optical transmission in thick TWCs [5,11]. Lamination is a common composite fabrication technique utilized to improve the bulk mechanical properties of single-ply anisotropic materials, like wood. Composites from two-dimensional, anisotropic plies can be rendered increasingly isotropic by layering plies at various orientations. Simultaneously optimizing the optical response in anisotropic laminates, however, remains a critical challenge for the field [11,12]. Progress has been made to improve haziness and to increase the thicknesses of TWCs through chemical modification of the intrinsically hydrophilic cellulosic phases to promote favorable chemical interactions with hydrophobic polymers [5]. Chemical compatibilization has improved internal surface wetting of the cellulosic phase and, thus, optical properties at larger length scales (3–10 mm) [5]. Interfacial modification of the cellulosic phase has also been shown to alter interfacial bonding, hygroscopicity, and coefficients of thermal expansion [13,14]. Cellulosic phases that have been chemically modified to have structurally similar moieties to the polymer matrix exhibited improved interfacial interactions and, thus, homogenization [12]. Given that chemically modifying cellulosic phases can have negative effects on the mechanical properties [15], tradeoffs between optical and mechanical properties are anticipated for chemically modified TWC laminates, posing yet another challenge to the field. 

To this end, the purpose of this work was to investigate the influence of chemical modification, namely acetylation, on the optical and mechanical properties of 1- and 2-ply TWCs with a commercial PMMA-based infiltrate. First, a delignified wood template was obtained from basswood sheets and infiltrated using a commercially available, photocurable PMMA-based resin either (1) as-is or (2) after modification via acetylation. The optical performance of the TWCs was investigated using Fourier transform infrared spectroscopy (FTIR) and ultraviolet-visible spectroscopy (UV-Vis). The mechanical properties of the TWCs were determined through tensile testing. Finally, the ability of classical lamination plate theory and a simple rule of mixtures to predict cross-ply tensile mechanical stiffness and strength, respectively, from ply-level mechanical properties was investigated and is reported herein.

## 2. Materials and Methods 

### 2.1. Materials

Longitudinally cut basswood (Tilia americana) veneers with a thickness of 1.6 mm and density of 0.41 g/cm^3^, produced by Midwest Products, were obtained from Home Depot (Boulder, CO, USA). Clear, photocurable methacrylate-based resin was obtained from Formlabs (RS-F2-GPCL-04). Sodium chlorite, sodium acetate, triethylamine (TEA), and acetic anhydride (AcAn) were obtained from Sigma Aldrich (St. Louis, MO, USA) and used without further purification. Acetic acid, ethanol, acetone, and dimethylformamide (DMF) were obtained from Fisher Scientific (Waltham, MA, USA) and used without further purification.

### 2.2. Composite Processing

#### 2.2.1. Production of a Delignified Wood Template

Basswood sheets were cut into 100 mm × 100 mm samples that were devoid of visual imperfections. Subsequently, the samples were oxidized to remove lignin at 80 °C with sodium chlorite at a concentration of 0.5 g/g of wood in a 1 N acetate buffer solution (sodium acetate and acetic acid to maintain a pH ~ 4.6). The oxidation proceeded until the sample had become visually white indicating that the delignified wood template had been successfully produced (~12 h). The delignified wood template was washed with hot deionized water three times to remove byproducts. The delignified wood template was then dehydrated via sequential solvent exchanges from ethanol to ethanol/acetone (1:1 by volume) to acetone. Each step of the solvent exchange was carried out twice for approximately 30 min. The delignified wood template maintained the same dimensions as the basswood sheet and was stored in acetone at ambient conditions until further use. 

#### 2.2.2. Base-Catalyzed Acetylation of Delignified Wood Templates

**Scheme 1:**
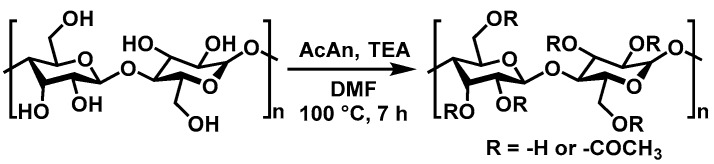
Cellulose acetylation with acetic anhydride (AcAn) catalyzed by triethylamine (TEA) in dimethylformamide (DMF). Hydroxyl groups on hemicelluloses are also accessible to this reaction, cellulose structure shown for simplicity.

Using the delignified wood template samples prepared in Section 2.2.1, a base-catalyzed acetylation was completed using AcAn and TEA in DMF, as shown in Scheme 1. The stoichiometric equivalents of reagents were calculated based on the assumption of theoretical hydroxyl (–OH) content of cellulose (54.05 g cellulose/mol –OH groups). The molar ratios of –OH:AcAn:TEA were 1.0:1.1:1.5 in an excess of DMF. The TEA and AcAn were added to the DMF then the delignified wood templates were immediately immersed in the reaction solution. The acetylation reaction proceeded at 100 °C for 7 h before the sample was removed from solution and sequentially washed with a series of DMF, then a 1:1 by volume DMF/acetone solution, and, finally, acetone. Each solvent exchange was carried out twice for approximately 30 min. The acetylated delignified wood template samples were stored in acetone until further use. Select samples were dried under dynamic vacuum at 50 °C for 2 hours to get masses and spectroscopic analysis (Section 2.3.1) for verification of acetylation.

#### 2.2.3. Laminate TWC Fabrication

One- and two-ply laminate TWCs were fabricated using the delignified wood template (C) and acetylated template (AC) samples and a clear, photocurable PMMA-based resin. To fabricate the laminate TWCs, acetone-saturated C and AC samples were submerged in the resin for a liquid–liquid exchange into the cellular structure. The submerged samples were then degassed at 70 °C for at least 12 hours until the wood cell structure was homogenously infiltrated with resin and acetone removed. The infiltrated C and AC samples were then placed between two glass plates and tightly secured with four medium (31.75 mm) binder clips. For 1-ply laminates, only a singular infiltrated template was used. For 2-ply laminates, two infiltrated templates were stacked, with one ply in the 0° direction (with respect to the fiber alignment) and the other ply oriented in the 90° (transverse to the fiber alignment) direction to create [0°/90°] TWCs. The assembly was placed in an oven at 70 °C under vacuum for 2 h for additional degassing at which point the internal white LED was illuminated for at least 6 h to cure the resin. Similar fabrication methods were used to generate samples of resin with the same thickness as 1- and 2-ply composites (termed R1 and R2). The clear resin was cast at similar thicknesses to ascertain differences in the optical response as a function of thickness. After fabrication, glass plates were carefully removed, and samples stored at ambient conditions until further testing could be performed. A list of the fabricated 1- and 2-ply laminate TWCs is provided in Table 1. 

### 2.3. Characterization of Laminate TWCs

#### 2.3.1. Fourier Transform Infrared Spectroscopy

Fourier transform infrared spectroscopy (FTIR) was conducted with a Cary 630 spectrometer (Santa Clara, CA, USA) equipped with an attenuated total reflection (ATR) accessory to substantiate the delignified wood template and modified acetylated templates. Eighty scans from ten locations on each substrate (C and AC) were collected and averaged upon acquisition for a representative trace for each material. Each spectrum was collected from 4000 to 500 cm^−1^ at a resolution of 1 cm^−1^. 

#### 2.3.2. Ultraviolet-Visible Spectroscopy

Ultraviolet-visible spectroscopy (UV-Vis) was carried out on a Cary 5000 UV-Vis-NIR Spectrophotometer (Santa Clara, CA, USA) equipped with a Diffuse Reflectance Accessory. Each specimen in Table 1 was tested, with the exception of the [90°], as the [0°] and [90°] samples are optically identical. Each scan was taken over the visible spectrum from 380–780 nm, using a BaSO_4_ reflectance standard at a rate of 5 nm/sec. In accordance with ASTM D1003, the percent transmittance and percent haze as a function of wavelength were calculated from four spectra per specimen: T1—baseline transmittance, T2—total sample transmittance, T3—inherent instrument scattering, and T4—total sample scattering [16]. Statistical significance of both transmittance and haze between C and AC samples were determined via ANOVA (analysis of variance) of the difference per unit wavelength between the respective 1- and 2-ply TWCs and is presented in Table 2.

#### 2.3.3. Tensile Mechanical Properties

**Figure 1:**
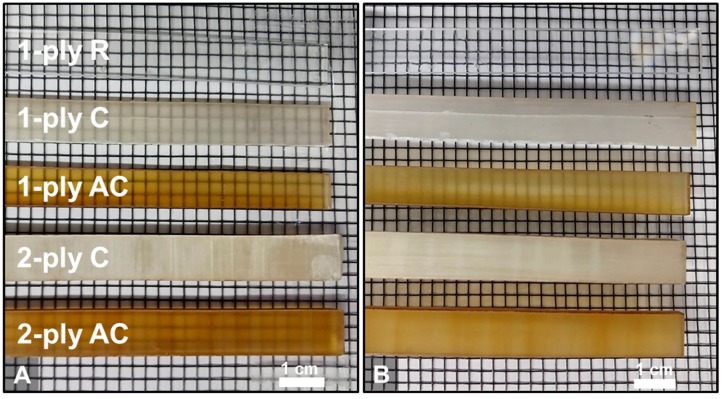
Images of selected TWC specimens demonstrating optical transparency. (**a**) The TWCs are flat on the grid and (**b**) the TWCs are raised 1 cm above the grid. (R—PMMA-based resin, C—delignified wood template, AC—acetylated delignified wood template).

After fabrication of the 1- and 2-ply TWCs and the pure resin (Sample R1), tensile test specimens were laser cut to 10 mm × 100 mm rectangular dimensions using an Epilog Legend 36EXT, as shown in Figure 1. The specimen nomenclature and property details for each class of prepared tensile specimens are provided in Table 1. Five replicate specimens for each class were tested to obtain tensile mechanical properties according to ASTM D3039 standard test methods [17]. Using a displacement-controlled rate of 2 mm/min, the tension tests were conducted using an Instron 5869 Universal Testing Machine (Norwood, MA, USA) and Micro-Measurements bonded strain gauges. For three specimens, strain was measured using a tee-rosette Micro-Measurements gauge (Item code: MMF307425) with a 350 Ohm grid resistance in order to compute Poisson’s ratio. For the remaining two specimens, strain was measured using a linear Micro-Measurements gauge (Item code: MMF307485) with a 120 Ohm grid resistance. 

Tensile strength and elastic modulus were determined for each specimen. The maximum tensile strength (MPa), $\sigma_{t\;max}$, was computed using the following equation:

(1) $$\sigma_{t\;max}=\frac{P_{max}}{A}$$

where $P_{max}$ is the maximum applied load (N) and A is the cross-sectional area (mm^2^) within the gauge length of the specimen. The elastic modulus (MPa), $E_{t}$, was computed using the following equation:

(2) $$E_{t}=\frac{\Delta \sigma_{t}}{\Delta \varepsilon_{l}}$$

where $\sigma_{t}$ is tensile stress (MPa) and $\varepsilon_{l}$ is strain (mm/mm) in the direction of loading. The tensile modulus, $E_{t}$, was computed by determining the slope of a linear regression based on the stress–strain data between 10% and 40% of the maximum tensile stress and the corresponding strain. Poisson’s ratio, υ, was determined for each specimen with a tee-rosette strain gauge according to the following equation:

(3) $$\upsilon =\frac{-\Delta \varepsilon_{t}}{\Delta \varepsilon_{l}}$$

where $\varepsilon_{t}$ is the strain in the direction orthogonal to the direction of loading. The Poisson’s ratio, υ, was computed by determining the slope of a linear regression based on the collected strain data between 15% and 45% of the maximum $\varepsilon_{l}$ and the corresponding $\varepsilon_{t}$.

#### 2.3.4. Mechanical Modeling of Elastic Modulus and Tensile Strength

Classical lamination plate theory was employed to predict the elastic modulus of a [0°/90°] laminate from ply-level properties obtained through mechanical testing. The compliance matrix, [S], is first defined as:

(4) $$\left[S\right]=\;\left[\begin{array}{ccc} \frac{1}{E_{1}} & \frac{-\nu_{21}}{E_{2}} & 0\\ \frac{-\nu_{12}}{E_{1}} & \frac{1}{E_{2}} & 0\\ 0 & 0 & \frac{1}{G_{12}} \end{array}\right]$$

in which *E*_1_, *E*_2_, *ν*_12_, and *ν*_21_ were measured directly and the in-plane shear modulus. *G*_12_ was neither experimentally measured nor calculated, given that its magnitude does not affect the prediction for [0°/90°] composite layups.

The compliance matrix must then be transformed, [T], into the orientation of testing for each ply, with the inclusion of the Reuter’s matrix, [R], to work around the 1/2 factor on shear components from classical definitions:

(5) $$\begin{array}{c} \left[T\right]=\;\left[\begin{array}{ccc} \cos^{2}\theta & \sin^{2}\theta & 2\cos\theta \sin\theta \\ \sin^{2}\theta & \cos^{2}\theta & 2\cos\theta \sin\theta \\ -\sin\theta \cos\theta & \sin\theta \cos\theta & \cos^{2}\theta -\sin^{2}\theta \end{array}\right]\\ \left[R\right]=\;\left[\begin{array}{ccc} 1 & 0 & 0\\ 0 & 1 & 0\\ 0 & 0 & 2 \end{array}\right] \end{array}$$

Utilizing [T] and [R], the transformed compliance matrix, $\left[\bar{S}\right]$, can be obtained for each ply and immediately obtain the transformed stiffness matrix, $\left[\bar{C}\right]$, to construct the ABD matrix:

(6) $$\begin{array}{c} \left[\bar{S}\right]=\left[R\right]{\left[T\right]}^{-1}{\left[R\right]}^{-1}\left[S\right]\left[T\right]\\ \left[\bar{C}\right]=\;{\left[\bar{S}\right]}^{-1} \end{array}$$

The constituents of the ABD matrix are defined as:

(7) $$\begin{array}{c} \left[A\right]=\;\overset{n}{\underset{k=1}{\sum }}\left[\bar{C}\right]\left(z_{k}-z_{k-1}\right)\\ \left[B\right]=\;\frac{1}{2}\overset{n}{\underset{k=1}{\sum }}\left[\bar{C}\right]\left(z_{k}^{2}-z_{k-1}^{2}\right)\\ \left[D\right]=\;\frac{1}{3}\overset{n}{\underset{k=1}{\sum }}\left[\bar{C}\right]\left(z_{k}^{3}-z_{k-1}^{3}\right)\\ \left[C^{*}\right]=\;\left[\begin{array}{cc} A & B\\ B & D \end{array}\right]\\ \left[S^{*}\right]=\;{\left[\begin{array}{cc} A & B\\ B & D \end{array}\right]}^{-1} \end{array}$$

The effective modulus, $E_{eff}$, for the cross-ply laminate can be calculated from $S_{11}^{*}$, which first needs to be adjusted for the total thickness (t) of laminate. $S_{11}^{*}$ is a component of the inverted [A] matrix and therefore has units of GPa^−1^ mm^−1^. By multiplying through by the thickness, the effective tensile modulus is determined to be:

(8) $$E_{eff}=\frac{1}{S_{11}^{*}t}$$

Monte Carlo simulations (*n* = 100,000) were performed on the experimental data of the model bounded by one standard deviation for sampling to add confidence to the predicted values (*n* = 100,000).

To model the tensile strength of the 2-ply TWCs, $\sigma_{\left[0{}^{\circ}/90{}^{\circ}\right]}$, a simple rule of mixtures was employed using strength data from the individual ply level using the equation:

(9) $$\sigma_{\left[0{}^{\circ}/90{}^{\circ}\right]}=\;V_{\left[0{}^{\circ}\right]}\sigma_{\left[0{}^{\circ}\right]}+V_{\left[90{}^{\circ}\right]}\sigma_{\left[90{}^{\circ}\right]}$$

where $\sigma_{\left[0{}^{\circ}\right]}$ and $\sigma_{\left[90{}^{\circ}\right]}$ are the tensile strengths and $V_{\left[0{}^{\circ}\right]}$ and $V_{\left[90{}^{\circ}\right]}$ are the volume contributions (both 0.5) of C and AC samples in the 0° and 90° directions, respectively [18]. All modeling input parameters are listed in Table 3. Confirmation between experimental data and computed predictions was performed using an unequal variances t-test.

## 3. Results and Discussion

### 3.1. Composite Processing

In processing TWC composites, sheets of longitudinally cut basswood veneers were delignified resulting in a mass loss and a color change to white as the lignin phase is decomposed, but a preservation of the interconnected cellular architecture of the wood as shown in other studies [2,3,11]. Subsequent filling of the cellular architecture of the delignified wood template with a PMMA-based resin serves to both provide mechanical reinforcement of the wood template as well as provide a refractive index matched medium by which photons can transmit through the filled wood template.

During delignification, a weight decrease of ~23% and complete loss of color was observed, indicating successful oxidation of the lignin. After the acetylation reaction, a weight percentage gain of ~6% was measured. The acetylation of the delignified wood template produced a brown color in solution and within the template that could not be removed, even with rigorous solvent washing. A byproduct, perhaps a salt complex of the triethylamine catalyst, may be responsible for the brown color throughout the substrate, seen in Figure 1, which has previously been reported in the literature [15]. According to Li et al., the color that manifests during the acetylation of the delignified wood template could be removed with an oxidation reaction [5]. The additional oxidation was not attempted on this system. Instead, the acetylated delignified wood template was processed into a TWC with the color retained. A variance in transmittance when placed on a grid and raised 1 cm above the grid can be seen for the fabricated composites (Figure 1). Despite the brown color, the underlying grid lines are qualitatively sharper for 1-ply AC and 2-ply AC when compared with 1-ply C and 2-ply C, respectively. Additionally, when raised 1 cm from the grid, the lines of the grid can only still be distinguished through the 1-ply AC and the 1-ply resin samples indicating a qualitative improvement of haze for the 1-ply AC sample relative to the 1-ply C sample.

### 3.2. Fourier Transform Infrared Spectroscopy

**Figure 2:**
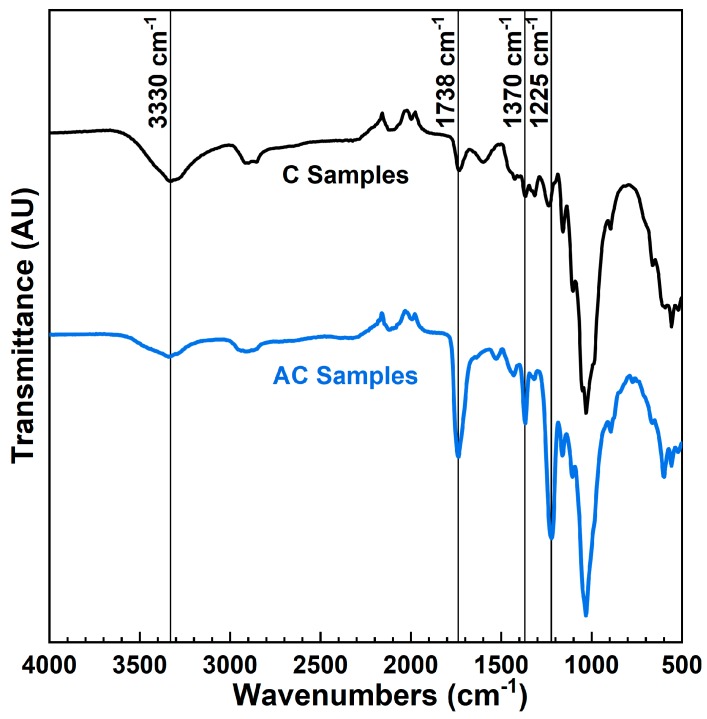
FTIR spectra of C and AC samples. Peaks correspond to –OH stretching (3300 cm^−1^), C=O stretching (1738 cm^−1^), C–H vibrational modes (1370 cm^−1^), and C–O stretching (1225 cm^−1^).

The C and AC substrates were analyzed via FTIR (Figure 2). The broad peak ~3300 cm^−1^ corresponds to –OH stretching. In the spectrum for delignified wood templates (C), the broad –OH peak is larger than the corresponding peak for the AC after the acetylation reaction, indicating that substitution at the hydroxyl groups occurred, as expected. The three sharper peaks at 1738 cm^−1^, 1370 cm^−1^, and 1225 cm^−1^ are more pronounced in the AC spectrum than in the C spectrum (Figure 2). Acetyl groups (–COCH_3_) contain strong signaling of motifs that are not typically present on the cellulosic chains [19]. The peak at 1370 cm^−1^ is the C–H bond vibration of non-hydroxyl substituted methyl groups, which serve to decrease the hydrophilicity of the cellulosic substrate [15]. The peak at 1225 cm^−1^ is the stretching mode of the C–O bond of the ester linkage formed on the cellulosic chain with the esterification of the alcohol [15]. The peak at 1738 cm^−1^ corresponds to the stretching mode of the C=O carbonyl group. In the C sample trace, a small peak centered on ~1738 cm^−1^ corresponds to low signaling of a carbonyl group, which can be attributed to decomposed lignin residue or oxidized chain end groups of hemicelluloses [20].

### 3.3. Ultraviolet-Visible Spectroscopy

**Figure 3:**
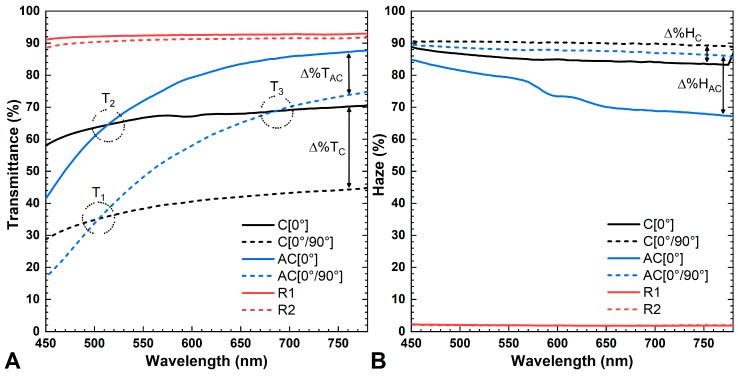
(**a**) Visible light transmittance and (**b**) haze for 1-ply (solid trace) and 2-ply (dashed trace) TWC composites and resin.

The percentage transmittance and percentage haze as a function of wavelength for each composite sample formulation is presented in Figure 3a and Figure 3b, respectively, and averages with ANOVA results are presented in Table 2. Measuring transmittance and haze in accordance with ASTM D1003 was independent of orientation for 1-ply TWCs, so orientation in the [0°] was arbitrarily chosen for reporting the overall transmittance and haze for the [0°] and [90°] single-ply TWCs. A drop off in transmittances at wavelengths <450 nm was observed, which is likely due to the resin photoinitiator absorption by 405 nm light tailing into longer wavelengths near the violet-blue border wavelength at 450 nm [21]. This drop-off is consistent across all samples. Therefore, for the purposes of analysis, only transmittance and haze measurements at wavelengths >450 nm are presented in Figure 3. 

As anticipated, R1 and R2 both exhibited high transmittances with minima of 91.1% and 88.5% and maxima of 93.0% and 91.8%, respectively. The average transmittance gap between R1 and R2 was 1.4 ± 0.3%. The transmittance for all TWCs had maxima in the upper red region (780 nm) of the visible spectrum and minima at 450 nm. C[0°] and C[0°/90°] had transmittance maxima of 70.6% and 44.9% and minima of 58.1% and 28.7%, respectively. The average gap in transmittance between C[0°] and C[0°/90°], Δ%T_C_, was 27.1 ± 1.3%. AC[0°] and AC[0°/90°] had transmittance maxima of 87.8% and 74.9% and minima of 41.5% and 17.0%, respectively. The average gap in transmittance between AC[0°] and AC[0°/90°], Δ%T_AC_, was 20.3 ± 4.8%. The AC samples had higher maxima than the C samples and lower minima in the case of each ply, but when comparing the gaps between 1-ply and 2-ply specimens, the AC samples had a smaller gap than the C samples (p-value < 0.05), suggesting that there was a lesser dependence on thickness when the delignified wood templates were acetylated. As expected, the brown color of the AC samples (Figure 1) encourages absorption in the green, blue, and violet region, causing the transmittance to decrease with wavelength at a faster rate than the C samples [22]. Changes in transmittance with wavelength for C and AC samples are denoted by the intersection points on the spectra at T_1_, T_2_, and T_3_. The intersection T_1_ occurs at 505 nm for the 2-ply systems and T_2_ at 515 nm for the 1-ply TWCs, above which the 1-ply and 2-ply AC samples exhibit higher transmittances and below which the C samples have the higher transmittances (Figure 3a). The intersection, T_3_, at 687 nm is notable, as it shows the point where AC[0°/90°] and C[0°] have equivalent transmittances, despite AC[0°/90°] having twice the thickness of C[0°].

The haze of a specimen is a measurement of light scattering. For high-clarity materials, a low haze is desirable. Haze in composites can occur from incompatibilities at the interface or discontinuities in the bulk phases that allow for air or void volume that can increase light scattering and reduce transmission. For single R1 and R2, the haze values were low, with the respective maxima of 2.3% and 2.2%, and minima of 1.8% and 1.9%. The gap in haze for R1 and R2 was effectively zero containing positive and negative difference values at various wavelengths resulting in an average difference of 0.02 ± 0.09%. The haze measurements for all TWCs from 780 nm to 450 nm followed the reverse trend to transmittance, with the highest haze values at 450 nm and the lowest haze at 780 nm, as can be seen in Figure 3b. C[0°] and C[0°/90°] had haze maxima of 88.9% and 90.8% and minima of 83.3% and 88.8%, respectively. The average gap in haze between C[0°] and C[0°/90°], Δ%H_C_, was 4.9 ± 1.1%. AC[0°] and AC[0°/90°] had haze maxima of 84.8% and 89.4% and minima of 67.2% and 85.6%, respectively. The average gap in haze between AC[0°] and AC[0°/90°], Δ%H_AC_, was 13.3 ± 5.0%. 

Statistical analysis confirmed that each haze spectrum is distinct from one another (p-value < 0.05) apart from R1 and R2 (p-value = 0.40). Each of the AC samples exhibited lower haze than the respective C samples (p-value < 0.05), suggesting improvement at the interface and, consequently, a reduction in photon scattering. The behavior of AC[0°] is notable, as the value for haze dropped the most across the visible spectrum (p-value < 0.05) to 67.2% at 780 nm. According to Li et al., acetylated TWCs of similar thickness demonstrated haze values down into 45–50%, though this system concerned a different wood species (balsa) and a different resin (pure PMMA, instead of a PMMA-based photocurable resin) [5].

### 3.4. Mechanical Properties

**Figure 4:**
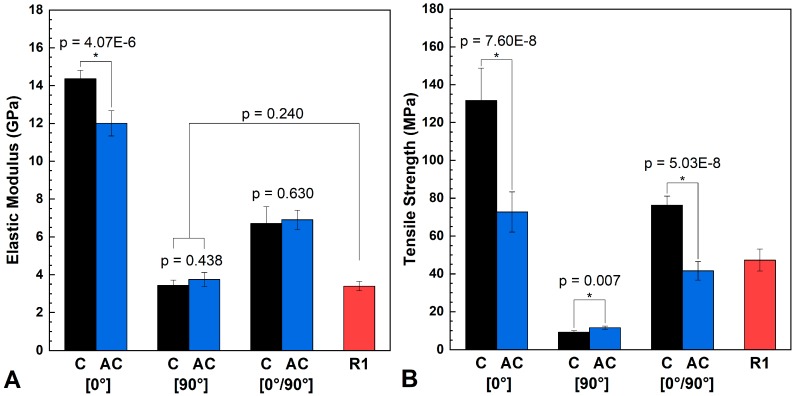
(**a**) Experimental tensile elastic moduli and (**b**) tensile strengths of C, AC, and resin (R1) samples (“p” denotes p-value for the conducted ANOVA).

The mechanical response of the TWCs is shown in Figure 4 and select numerical data can be found in Table 3 and Table 4. In Figure 4, it is evident that the tensile modulus in the [0°] orientation was reduced for the AC samples. In the [90°] and [0°/90°] orientations, however, the same decrease for AC samples is not observed. It is notable that the modulus of the resin (R1) is similar to that of the [90°] C and AC samples. Anisotropic fibrous materials, such as wood, have high stiffness in the fiber (i.e., longitudinal) direction, often requiring compositing or lamination in order to improve the bi-directional properties of the material. Removal of lignin from wood is known to reduce the mechanical integrity of the resulting template. However, filling in the cellular architecture with a stiff resin can restore tensile stiffness [7]. Two-way ANOVA for elastic modulus of all TWC formulations indicated that there were main effects of specimen type (C and AC samples) and orientation (0°, 90°, and 0°/90°), with an interaction between the two (p-value < 0.05). Comparisons of the C and AC samples at specific orientations showed that, in the 0° orientation, the difference between samples was significant (p-value = 4.07 × 10^−6^). In the 90° and the 0°/90° orientations, however, the difference in stiffness was not statistically significant (p-value = 0.438 and 0.630, respectively). An additional one-way ANOVA determined that the elastic modulus for C[90°], AC[90°], and resin were not statistically different from each other (p-value = 0.240). This similarity indicates that the contribution of stiffness response in the 90° orientation is likely due to the resin only. 

Similar to the stiffness response, tensile strength exhibited a trend of orientation dependence with the [0°] samples giving higher strengths along the fiber direction. Between the C and AC samples, however, there was a more pronounced decrease for the [0°] and [0°/90°] for the AC samples. There was not an observed decrease for the AC samples in the [90°] orientation, though both of the [90°] samples were weaker than any other TWC and even the resin due to facile fiber separation in that applied load direction. With two-way ANOVA for the tensile strength, the dependent variables were log-transformed to satisfy ANOVA assumptions of normality and homoscedasticity. In this analysis, there were main effects of specimen type (C and AC samples) and orientation (0°, 90°, and 0°/90°), with an interaction between the two main effects (p-value < 0.05). Comparisons of the C and AC samples at specific orientations showed that there was statistical significance between all orientations and tensile strengths, with C[0°] and C[0°/90°] being larger than the AC samples at the same orientations (p-value = 7.60E-6 and 5.03E-8, respectively). AC[90°] had a strength advantage over C[90°] (p-value = 0.007), suggesting an improvement of interfacial bonding between AC and the resin. 

Two-way ANOVA for Poisson’s ratio of all TWC formulations indicated that there were neither statistically significant main effects of specimen type nor interactions between specimen type and orientation (p-value > 0.05). The only main effect on Poisson’s ratio was sample orientation, as expected, with samples tested in the 90° being different from the 0°, and 0°/90° samples being statistically similar (p-value < 0.05). 

When specifically comparing chemical treatments at varying orientations, acetylation influenced the stiffness and strength of the resulting TWCs. Along the fiber direction (0°), acetylation had the largest effect on the mechanical properties, with the stiffness and strength of AC[0°] samples being significantly reduced relative to C[0°]. This reduction is likely due to the disruption of the hydrogen bonding of cellulose and hemicellulose chains through substitution with hydrophobic acetyl moieties. In the 90° orientations, no statistical difference in stiffness exists between C[90°], AC[90°], or resin samples, but the tensile strength of AC[90°] was greater than C[90°], but both were greatly reduced compared to the resin. When applying a load orthogonal to the alignment of cellulosic fibers, it was expected that a weaker mechanical response would be observed. The stiffness of the 90° specimens were statistically similar to the resin, suggesting that the resin matrix around the fibers was largely responsible for the stiffness under the applied load. Additionally, the homogenous resin exhibited a higher strength relative to either 90° TWC, which is likely a result of critical flaw sizes within the TWCs, resulting in diminished strength responses. Interestingly, AC[90°] was stronger than C[90°], meaning that the acetylation of the delignified wood template likely improved the interfacial interactions between the template and resin matrix. 

In the cross-ply system, the stiffness was similar between C and AC samples, but C[0°/90°] had a higher strength than AC[0°/90°]. Following the individual ply-level system, the 0° direction was the strength-dominating orientation, meaning that the C samples were expected to be stronger relative to AC samples. The stiffness response of C[0°/90°] and AC[0°/90°] were not statistically different (p-value = 0.630), which was identical to the trend of the stiffness response in the 90° TWC samples. This result indicates that, despite the reduced stiffness of AC[0°] relative to C[0°], the addition of a 90° ply homogenizes the elastic response in these 2-ply TWCs. 

### 3.5. Mechanical Modeling

Using the 1-ply mechanical response data from Table 3, the prediction for the 2-ply elastic response was calculated using classical lamination plate theory and the tensile response was calculated using the rule of mixtures. The predicted and experimental responses are shown in Table 4. The prediction of the tensile strength by Monte Carlo simulation (*n* = 100,000) was in relatively good agreement for all samples with difference in means of 5.9 MPa and 0.5 MPa for C[0°/90°] and AC[0°/90°], respectively. The difference in means for the stiffness response was found to be 0.29 GPa and 0.64 GPa for C[0°/90°] and AC[0°/90°], respectively.

Using an unequal variances t-test the prediction of elastic modulus for the C[0°/90°] and AC[0°/90°] TWCs were found to be similar to the experimentally obtained values (p-value = 0.50 and p-value = 0.07, respectively). The predicted and experimental tensile strength of the 2-ply TWCs were also determined to be statistically similar for the C[0°/90°] and AC[0°/90°] TWCs (p-value = 0.053 and p-value = 0.838, respectively). The variance of the predicted tensile strength increased relative to the experimental values when subjected to a Monte Carlo simulation, while the variance in predicted elastic modulus did not. This increase in variance for strength is likely a result of the disparity in magnitude of the standard deviations of the input tensile strengths (Table 3). For $\sigma_{\left[0{}^{\circ}\right]},$ the standard deviations were >10 MPa, while for $\sigma_{\left[90{}^{\circ}\right]}$, they were <1 MPa, which could contribute to a larger variance in a Monte Carlo simulation. The model using classical lamination plate theory was able to successfully predict the effective elastic modulus for both C[0°/90°] and AC[0°/90°]. The larger experimental deviation from the predicted stiffness output for AC[0°/90°] was attributed to the statistically lower (AC[0°]) or indistinguishable (AC[90°]) elastic moduli compared to C samples in the 1-ply orientation. This model does not account for the adhesion interactions of the cross-plied sheets infiltrated with resin. If the interaction between the AC substrate and the resin were improved, the interface between the cross-plied sheets could be stronger than expected.

### 3.6. Mechanical–Optical Response

**Figure 5:**
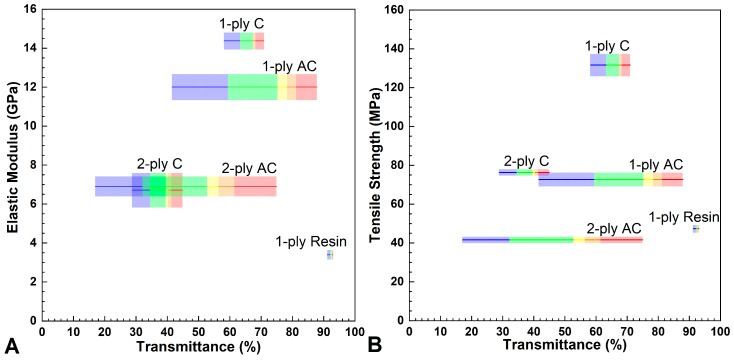
Comparison of the optical transmittance to (**a**) elastic modulus and (**b**) tensile strength to visualize the how each TWC is transmitting light across the visible spectrum. Mechanical data for the 1-ply samples utilized the [0°] properties. Thicknesses—1-ply C: 1.63 mm, 1-ply AC: 1.66 mm, 1-ply resin: 1.30 mm, 2-ply C: 3.18 mm, 2-ply AC: 3.42 mm.

When considering infrastructure materials that serve optical purposes (e.g., windows), it is desirable to understand the correlation, if any, between optical and mechanical responses. As a reference, soda-lime glass, commonly used for windows, has an average transmittance of ~90% across the visible light spectrum, an elastic modulus between 68–72 GPa, and a tensile strength between 31–35 MPa [23,24]. Although the TWCs presented here have singular mechanical response values, there is a strong dependence of wavelength on the optical response. Instead of reporting transmittance and haze values at single wavelengths, Figure 5 shows the total span of transmittance values for each specimen with the spectral color shown. Similar to the discussion of the UV-Vis traces, wavelengths <450 nm are omitted (no violet) and all 1-ply samples simplified to the [0°] notation as the optical measurements were not orientation dependent. For every specimen, the maximum transmittance values were at the highest recorded wavelength (780 nm—red) and declined with wavelength. The color segments shown are effectively the point-to-point decreasing slope for each region of color in the visible spectrum projected on a line. The solid central line corresponds to the mean elastic moduli or tensile strength for that specimen and the surrounding translucent bar is the first standard deviation. A rationale behind presenting the projection of optical response is due to the multiple intersections, T_1_, T_2_, and T_3_, in Figure 3a, where superior optical performance of AC vs. C samples is wavelength dependent. 

Figure 5 graphically illustrates the ideal design and performance targets for TWCs, namely high stiffness and strength coupled with high, narrowly distributed transmittance values. As expected, the resin satisfies the high, narrowly distributed transmittance, but falls short of good mechanical properties when compared to the TWCs. Both C[0°] and C[0°/90°] have narrower transmittance distributions and higher strengths than either AC[0°] or AC[0°/90°], but do not achieve higher maximum transmittances. It is evident that, while further development of TWCs is required to push the bounds and achieve a mechanically robust and high transmittance composite material, these plots serve as useful assessment tools for evaluating high-performance TWCs that seek to maximize both optical and mechanical performance.

## 4. Conclusions

This study investigated the optical and mechanical response of acetylated delignified wood template TWCs and the applicability of conventional mechanical models to predict multi-ply mechanical response from ply-level properties. Results substantiate that acetylation of the delignified wood template influenced both the mechanical and optical responses of TWCs when compared to the unmodified delignified wood template. The optical transmittance and haze were improved for acetylated TWCs compared to unmodified TWCs at longer wavelengths. The brown coloration that manifested within the acetylated TWCs reduced the transmittance at lower wavelengths. There was a reduction in the transmittance gap and an increase in the haze gap between 1- and 2-ply AC TWCs, indicating that the thickness dependent optical response was improved upon interfacial modification. There was a reduction in the stiffness and tensile strength in the [0°] upon acetylation, but when cross-plied, the AC[0°/90°] had a similar stiffness to C[0°/90°]. This reduction in stiffness and strength is likely due to the disruption of hydrogen bonding in the celluloses and hemicelluloses upon modification. However, evidence that the acetylation improved the interfacial binding between the substrate and the resin came in part from the fact that AC[90°] was stronger than C[90°]. The modulus of AC[0°] was lower than C[0°], while the elastic moduli between C and AC samples in the [90°] and [0°/90°] were statistically similar to one another, suggesting an improvement in interaction with the resin when the substrate was acetylated. Prediction of the elastic moduli using classical lamination plate theory and tensile strengths using a simple rule of mixtures showed good agreement with experimental data, indicating the applicability of these mechanical models to predict the multi-ply behavior of TWCs using ply-level mechanical properties. 

## Figures and Tables

**Table 1:** 1- and 2-ply TWC laminate formulations.

Specimen Nomenclature	Property				
	Number of Plies	Thickness (mm)	Fiber Orientation	Fiber Reinforcement	Polymer Matrix
C[0°]	1	1.55 ± 0.02	0°	Delignified Basswood Template	PMMA-based Resin
C[90°]	1	1.72 ± 0.04	90°		
C[0°/90°]	2	3.18 ± 0.05	0°/90°		
AC[0°]	1	1.66 ± 0.02	0°	Acetylated Delignified Basswood Template	
AC[90°]	1	1.67 ± 0.03	90°		
AC[0°/90°]	2	3.41 ± 0.06	0°/90°		
R1 (Resin Only)	N/A	1.30 ± 0.03	N/A	N/A	
R2 (Resin Only)	N/A	3.11 ± 0.10			

**Table 2:** Transmittance and haze responses with ANOVA results.

Ply	Response	C	AC	R	ANOVA–Sample Type
1	Average Transmittance	66.9 ± 3.0%	75.6 ± 12.7%	92.5 ± 0.4%	p < 0.05
2		39.8 ± 4.2%	55.3 ± 17.0%	91.0 ± 0.7%	p < 0.05
ANOVA–Ply level		p < 0.05	p < 0.05	p = 0.03	−
Δ%T		27.1 ± 1.3%	20.3 ± 4.8%	1.4 ± 0.3%	p < 0.05
1	Average Haze	85.1 ± 1.4%	74.4 ± 5.8%	1.9 ± 0.1%	p < 0.05
2		90.0 ± 0.5%	87.7 ± 0.9%	2.0 ± 0.1%	p = 0.0001
ANOVA–Ply level		p < 0.05	p < 0.05	p = 0.40	−
Δ%H		4.9 ± 1.1%	13.3 ± 5.0%	0.02 ± 0.09%	p < 0.05

* “p” denotes “p-value” for the conducted ANOVA.

**Table 3:** Experimental results and input parameters for the mechanical models.

Model	Parameter	Value	Units	Description
Classical Plate Lamination Theory for Elastic Modulus	$E_{1}$	C: 14.4 ± 0.4	GPa	Elastic Modulus in the [0°] orientation
		AC: 12.0 ± 0.7		
	$E_{2}$	C: 3.43 ± 0.29	GPa	Elastic Modulus in the [90°] orientation
		AC: 3.75 ± 0.37		
	$\nu_{12}$	C: 0.375 ± 0.016	-	Poisson’s ratio measured from the [0°] orientation
		AC: 0.365 ± 0.016		
	$\nu_{21}$	C: 0.119 ± 0.011	-	Poisson’s ratio measured from the [90°] orientation
		AC: 0.098 ± 0.003		
	h	C: 3.18 ± 0.05	mm	Composite Thickness
		AC: 3.41 ± 0.06		
Rule of Mixtures for Tensile Strength	$\sigma_{\left[0{}^{\circ}\right]}$	C: 131.6 ± 17.1	MPa	Tensile Strength in the [0°] orientation
		AC: 72.7 ± 10.7		
	$\sigma_{\left[90{}^{\circ}\right]}$	C: 9.11 ± 0.75	MPa	Tensile Strength in the [90°] orientation
		AC: 11.49 ± 0.88		

**Table 4:** Predicted elastic modulus and experimental mechanical response for 2-Ply TWC laminates.

2-ply TWC	$E_{exp}(\mathrm{GPa})$	$E_{pred\;}(\mathrm{GPa})*$	CLPT Model Validation	$\sigma_{exp}(\mathrm{MPa})$	$\sigma_{pred}(\mathrm{MPa})*$	RoM Model Validation
C[0°/90°]	6.71 ± 0.89	6.42 ± 0.33	p = 0.630	76.3 ± 4.9	70.4 ± 8.5	p = 0.053
AC[0°/90°]	6.90 ± 0.51	6.26 ± 0.39	p = 0.070	41.6 ± 4.9	42.1 ± 5.3	p = 0.838

* Modulus from classical lamination plate theory (CLPT) and strength from the rule of mixtures (RoM) with Monte Carlo simulations (*n* = 100,000), “p” denotes p-value for unequal variance t-test.
